# 163. MIC Shifts in Response to Increased Antibiotic Utilization During COVID-19 Pandemic

**DOI:** 10.1093/ofid/ofab466.365

**Published:** 2021-12-04

**Authors:** Jonathan Byrd, Neena Thomas-Gosain, Jane V Eason, Jessica Bennett, Jarred Bowden

**Affiliations:** 1 UTHSC, Memphis, Tennessee; 2 University of Tennessee Health Science Center, Memphis, Tennessee; 3 Memphis VA Medical Center; UTHSC, Memphis, Tennessee; 4 VAMC Memphis, Germantown, Tennessee; 5 Memphis VA Medical Center, Memphis, Tennessee

## Abstract

**Background:**

Multiple studies have shown that antibiotic utilization increased during the COVID-19 pandemic. However, the impact of this increased utilization has not been well established. The aim of this study is to describe the trends in minimum inhibitory concentrations for various antibiotics against common gram-negative pathogens observed since the start of the COVID-19 pandemic as compared to previous years.

**Methods:**

This retrospective study was conducted at the Memphis VA. All respiratory, urine, and blood culture MicroScan results run from October 2017-March 2021 were analyzed. Only inpatient and emergency department data was included. The MIC50 and MIC90 of seven antibiotics for four of the most common pathogens were trended by quarterly intervals.

**Results:**

MIC50 and MIC90 were compared using standardized breakpoints. As compared to previous years, *Pseudomonas aeruginosa* was noted to have the most sustained increase in MIC90 across various antibiotics. In the last 3 quarters of the study time frame, piperacillin-tazobactam mean MIC90 increased from 32 to 64, cefepime from 8 to > 16, and meropenem from 4 to > 8. *Escherichia coli* had a sustained increase in ceftriaxone MIC90 from < 1 to > 8 in the final quarter of 2020 and beginning of 2021. *Klebsiella pneumonia* was also found to have a sustained increase in cefepime mean MIC90 from < 1 to > 16 during the year of 2020, with return to previous MIC90 the following quarters.

**Conclusion:**

Previous studies have clearly demonstrated a widespread increase in antibiotic utilization during the COVID era. Our study demonstrates how even short-term increases in antibiotic use can lead to shifts in MIC, if not outright resistance. This was demonstrated across multiple common gram-negative pathogens and to various broad-spectrum antibiotics which were commonly used more frequently during COVID-19. Further analysis will be needed to determine whether these trends continue or whether the decrease in antibiotic utilization in the recent months will lead to similar decrease in MIC.

**Disclosures:**

**All Authors**: No reported disclosures

